# Clinical-Epidemiological Characteristics of Hidradenitis Suppurativa: A Retrospective Cohort Study from a Tertiary Care Centre in Northern Israel

**DOI:** 10.3390/jcm12123921

**Published:** 2023-06-08

**Authors:** Anan Hammud, Emily Avitan-Hersh, Ziad Khamaysi

**Affiliations:** 1 Department of Dermatology, Rambam Health Care Campus, Haifa 3109601, Israel; ananhammoud@gmail.com (A.H.); emily@technion.ac.il (E.A.-H.); 2 The Ruth and Bruce Rappaport Faculty of Medicine, Technion, Israel Institute of Technology, Haifa 3109601, Israel

**Keywords:** hidradenitis suppurativa, adalimumab (Humira), Hurley stages, Arabs and Jews

## Abstract

Background: Hidradenitis suppurativa (HS) is characterised by inflamed lesions that typically appear in apocrine-rich flexural areas. Although studies have reported clinical and epidemiological data from western countries, data from the Middle East are scarce. The aim of this study is to characterise the differences in the clinical characteristics of patients with HS of Arab and Jewish ancestry and review the clinical characteristics, the course of the disease, the comorbidities, and the response to treatment. Methods: This is a retrospective study. We collected clinical and demographic data from patient files between 2015–2018 at the Rambam Healthcare Campus dermatology clinic—a tertiary hospital located in the north of Israel. Our results were compared to those of a previously published Israeli control group registered in Clalit Health Services. Results: Of the 164 patients with HS, 96 (58.5%) were men and 68 (41.5%) were women. The average age at diagnosis was 27.5 years and the average latency between the onset and diagnosis of the disease was 4 years. We found a higher adjusted prevalence of HS in Arab patients (56%) than in their Jewish counterparts (44%). Gender, smoking, and obesity, as well as axilla and buttock lesions, were risk factors for severe HS, with no differences between ethnicities. No differences were documented in comorbidities and in response to adalimumab, with a high overall response rate of 83%. Conclusions: Our findings revealed differences between Arab and Jewish patients with HS in terms of incidence and gender predominance, while no differences were documented in comorbidities and response to adalimumab.

## 1. Introduction

Hidradenitis suppurativa (HS) is a dermatologic condition characterised by inflamed lesions which appear in flexural areas rich in apocrine glands [1]. The onset of the disease is usually after puberty and is most common during the third decade of life [2].

The disease is underdiagnosed in many countries, including Israel. Its prevalence ranges from 0.053% in some countries to 4% in others, an indication of under-diagnosing [3]. HS is considered more common in women, with a ratio of 3:1 between the genders [1].

There are several contributors to the pathogenesis of HS, such as genetic predisposition and immune dysregulation. A recent study has shown that up to 34% of patients with HS had a positive family history [4]. In addition, there is a dysregulation of the inflammatory response, including both innate and acquired immune responses [3]. Bacterial infection is also considered to play a role in the pathogenesis of HS [5].

The severity of HS disease is classified by the Hurley grading system, which classifies HS into three groups according to the presence of abscesses and sinuses. Involvement of multiple areas with scarring and infected subdermal fistulas may lead to functional disturbances [6]. The burden is further intensified by multiple comorbidities, some of which are common such as smoking, metabolic syndrome (including hypertension, hyperlipidaemia and excess weight) in 40% of the patients [7], polycystic ovary syndrome in 38% [8], malignancy in up to 50% [9,10], depression in up to 42% [11], and inflammatory bowel disease in 17–38% [12].

Treatment of HS is challenging and includes a wide range of topical and systemic antibiotics [13,14], hormone therapy [15], oral retinoids [16], corticosteroids, and biological treatments, mainly anti-TNF alpha [17,18]. Additional options are surgical and laser therapies [19,20,21].

A previous epidemiological study on HS patients from Israel [22] analysed patients treated in the community setting (Clalit Health Services) and was based on digitally annotated data.

The purpose of this study is to characterise the differences in clinical characteristics of Israeli patients with HS of Arab and Jewish origin, and to review their clinical characteristics, disease course, risk factors, and different treatment options.

## 2. Materials and Methods

This study is a retrospective observational study in which we gathered clinical, demographic, and laboratory results from the computerised files of 219 patients diagnosed and treated for HS at Rambam Healthcare Campus dermatology clinic—a tertiary hospital located in the north of Israel—between 2015 and 2018. Patients with missing data (n = 55) were excluded, resulting in a total of 164 patients included in this study.

Israel has two main ethnic groups: Jews and Arabs. The primary investigator determined patients’ ethnicities based on their names, language spoken, and personal meetings during examinations.

The diagnosis was determined by a single dermatologist, based on clinical characteristics, medical history, and physical examination of each patient. Each patient’s age, sex, smoking history, age at diagnosis, duration of the disease, diagnostic delay, location of the lesions, and severity of the disease were recorded according to their Hurley stage. We also recorded treatment and family history, body mass index (BMI), associated cutaneous and systemic diseases, and other comorbidities. A positive family history was determined if patients reported first- or second-degree relatives with HS.

The evaluation of the treatment response was classified into four groups:

(1) Complete remission: improvement of 100%.
(2) Significant response: improvement of 50% or more (but not 100%).
(3) Mild response: improvement of less than 50 % (but not 0%).
(4) No response: improvement of 0%.

The determination of obesity was classified according to BMI (equal or greater than 30). Smoking history was classified as past or current smoker or non-smoker. Hypertension and type 2 diabetes were determined according to common clinical practice.

Demographic data were compared with the general population according to 22,085 patient files registered with Clalit Health Services [22].

### Statistical Analysis

Variation of treatment response is shown according to sub-categories (gender, overweight, hypertension, etc.) and calculated using the Pearson Chi square.

Treatment response for quantitative measurements (age and duration till diagnosis) was conducted using ANOVA or Kruskal–Wallis test. Data processing was conducted using SPSS 25. Significance was shown when *p* < 0.05.

Descriptive statistics in terms of mean, standard deviation, percentage, median, and percentiles were calculated for all parameters in the study. Differences between the data from the Rambam Medical Centre versus data from the literature were tested using the Fisher exact test or *t*-test. Differences between three groups (complete remission + significant, mild, and no effect) were tested by Kruskal–Wallis with an adjustment for multiple comparisons; *p* < 0.05 was considered as significant. SPSS program was used for all statistical analyses.

## 3. Results

Characteristics of the patients are presented in Table 1. Of the 164 patients with HS, 96 (58.5%) were men and 68 (41.5%) were women. The average age at the first visit was 33.9 years (range 11–75, 33.9 ± 13.8 years). The average age of onset was 27.5 years. Consequently, the interval between the onset and diagnosis of the disease was 4 years. While most of the patients (68.1%) presented between 10 and 39 years, 42% of the male patients had an average age of onset equal to or above 40 years, compared to only 28% of the females (Table 2).

**Figure 1:**
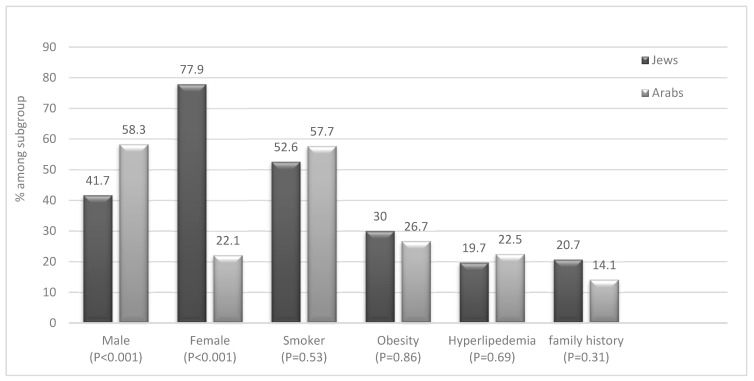
Disease characteristics of HS patients according to ethnicity.

Most of the patients (57%) were Jews while the remainder (43%) were Arabs. However, when we adjusted the prevalence according to the total visits to the clinic during the same period, we obtained a higher adjusted prevalence of HS in Arab patients rather than in Jewish patients (56% vs. 44%, respectively). Additionally, the majority (57%) of Jewish patients with HS were females (*p* < 0.001) while the opposite was observed among Arab patients (Figure 1). This indicates a significant difference in the predominance of the male gender in the HS population compared to the Jewish one.

Eighteen percent of all patients had a family history of HS, which was far more common among Jewish patients, 20.7%, compared to the Arab patients, 14% (Figure 1).

When we compared the clinical data of patients in this study (n = 164) with data derived from the general population cohort in Israel (Table 3) registered at Clalit Health Services [22], (n = 22,085), we found that the percentage of Arabs was 4 times higher in our cohort than in the general population.

### 3.1. Associated Diseases

**Figure 2:**
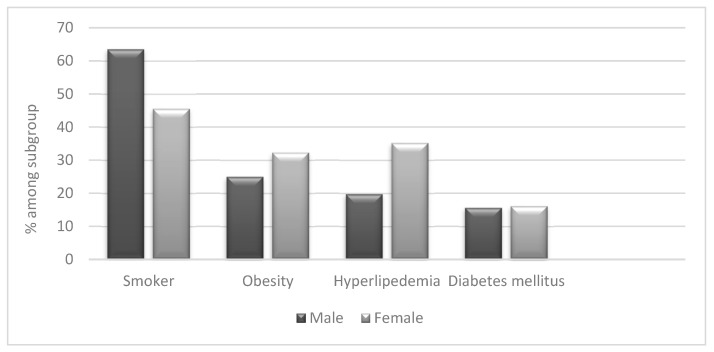
Disease characteristics of HS patients according to gender.

When analysing co-morbidities, we did not find significant differences between Arab and Jewish patients with HS (Figure 1 and Figure 2). We found a high prevalence of obesity (28%) in HS patients compared to the general population (7%). However, the prevalence of hypertension and diabetes was similar among HS patients and controls. Furthermore, 55% of the patients were active or past smokers, among them 63.5% men, compared to 12.9% of the general population (Table 3); 57.7% of Arab patients with HS and 52.6% of Jewish patients are (or were) smokers (Figure 1).

The two most common skin comorbidities were eczema (12%) and acne (11%). Less common were psoriasis and other diseases such as non-HS-related SCC (squamous cell carcinoma), drug allergies, burns, and skin infections.

Affected areas—The areas most frequently affected were the groin (74%), followed by the axilla (63%), buttocks (43%), and sub-mammary folds (27%). When analysed by gender, genital lesions were more common in males; 56% of the patients with groin lesions and 66% of the patients with buttock lesions were men; 50% of the patients with axillary lesions and 60% of the patients with sub-mammary lesions were females. There were no differences in affected areas between Arabs and Jews.

### 3.2. Diagnostic Factors for Predicting Disease Severity according to Hurley Stages

**Figure 3:**
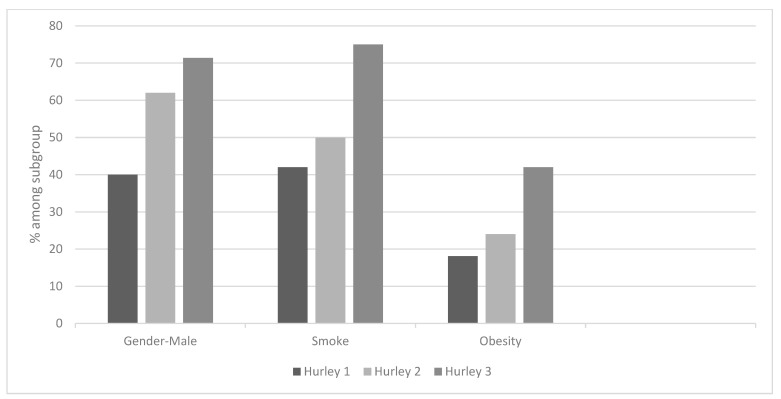
Disease characteristics and their relationship with disease severity according to Hurley classification.

Hurley stage I was seen in 20%, stage II in 51%, and stage III in 29% of the patients. Males had a higher risk factor for more severe disease: 62% of patients classified with Hurley II and 71.4% of patients classified with Hurley III were men (Figure 3). Smoking and obesity were also found to be risk factors for more serious disease: 50% of Hurley II and 75% of Hurley III patients were smokers, 24% of Hurley II and 42% of Hurley III patients were overweight.

**Figure 4:**
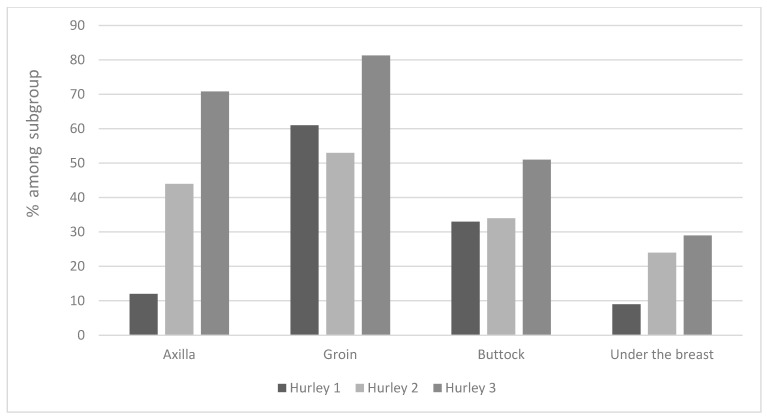
Lesion location and its relationship with disease severity according to Hurley classification.

Regarding affected areas (Figure 4)—Axillary and buttock lesions were found to predispose to a more severe disease: 34% of Hurley II and 51% of Hurley III patients presented buttock lesions; 44% of Hurley II and 70.8% of Hurley III patients had axillary lesions.

No correlation was found between family history, ethnicity, and disease severity.

### 3.3. Treatment Response

Our cohort was treated in a tertiary medical centre; therefore, most patients had already failed several treatment modalities upon referral. These mainly included short courses in oral antibiotics and topical treatment.

There was no difference in response to treatment between Arabs and Jews.

Topical antibiotic—Almost all patients received topical treatment in combination with oral antibiotic, making this modality of treatment difficult to assess.

Oral antibiotic treatment—The most common antibiotic used was clindamycin (128 patients); 21% of the patients showed significant improvement in response to this treatment.

The second most common antibiotic was rifampin (62 patients), which was administered mainly in combination with clindamycin.

Prednisone treatment—Several patients were treated with oral prednisone (n = 24) and most of them had a short-term partial response.

Retinoids treatment—In response to acitretin treatment (n = 27), 22% of patients showed significant improvement, although most patients only showed partial response with recurrence of the disease. Isotretinoin (n = 40) was indicated primarily in patients with a co-occurrence of acne and showed partial improvement in 25% of treated patients; 65% of treated patients were classified as non-responsive to treatment.

Surgical treatments—Data are presented in Table 4. The most common surgical procedure was abscess drainage. The response to treatment was temporary and most of the patients had recurrence. A minority of patients underwent a broad excision procedure and showed signs of recurrence at the same site. Other surgical treatments were also recorded. Seven patients were treated with an Er:YAG laser using a deroofing method to treat inflamed sinuses, all of whom showed healing of the treated areas.

Adalimumab patients who did not receive several treatment options were selected for biological treatment with adalimumab (n = 36): 8% fully responded, 33% of patients markedly improved, 42% had partial improvement, and 17% did not respond. Patients who did not respond to adalimumab were treated with infliximab (n = 5). Of these, one patient had a complete response, one had marked improvement, two had partial improvement, and one patient did not have a therapeutic response.

Prognostic factors of the disease and response to adalimumab treatment—We searched for parameters that would predict treatment response in patients treated with adalimumab (Table 5). Most parameters including ethnicity, gender, Hurley stage, age, hypertension, excess weight, hyperlipidaemia, smoking, leukocyte count, and liver function test were found to have no influence on treatment response. Two parameters were time to diagnosis and location of lesion involvement (groin, axilla, buttocks, and sub-mammary) which had a significant effect in predicting the response to adalimumab treatment. Although a better response was observed in patients with a shorter interval until diagnosis (*p* = 0.049), a worse response was observed when lesions involved the buttocks (*p* = 0.037). All patients who did not respond to adalimumab had buttock lesions, compared to 40% of patients who had a partial response and 47% of patients who had marked improvement.

## 4. Discussion

In this single-centre study, we included 164 patients, both Arabs and Jews, with HS who were diagnosed and treated by the same dermatologist. We investigated the severity, risk factors, and demographic, clinical, and treatment characteristics of HS. Although there are several studies in western countries, epidemiological data for patients from the Middle East and Asia is rare [23]. Recently, a study held in Israel described the demographic and clinical characteristics of HS patients extracted from the Clalit Health Services database [22]. This study was carried out in an ambulatory setting (community) and was based on digitally annotated data. On the other hand, our study was conducted in a single centre, in which we examined all patient files, the treatment regimen was consistent between patients, and all patient diagnoses and comorbidities were validated.

We documented a high proportion of Arabs among HS patients: 56% of our cohort. This is compatible with the findings by Shalom et al. [22]. In their study, 25% of HS patients were Arabs compared to only 11% Arabs in the control population. The over-representation of the Arab population among HS patients may suggest the effect of environmental factors (nutrition, smoking) which could be affected by their lower socio-economic situation; genetic predisposition could be another explanation. In fact, a slightly higher prevalence of smoking was found among our Arab patients with HS.

HS disease in our study was found to be more prevalent in men (58%). This difference was prominent among the Arab population where the incidence in males was 77.4%. On the other hand, in the Jewish population, the majority of patients were women (52.7%). The findings in the Arab population contradict previously reported data suggesting a higher prevalence of HS in women. Shalom et al. also showed female predominance (60%) [22]. This difference is probably due to a selection bias of the more severe patients referred to our tertiary centre (suggested by the high percentage of Hurley II and III patients (80%) (Table 1). In particular, a male predominance was reported in more severe stages of the disease in other cohorts [8].

The average age of onset was higher in men than in women (Table 2), which corresponds to previous reports [24]. This is most probably due to the differences in hormonal profiles between men and women. Additionally, the mean age of diagnosis was 39.9 years, which corresponds to the mean age found in the study conducted by Guy Shalom et al. (38.5 years) [22]. The mean age of diagnosis found in Israel is higher than the age of diagnosis found in other cohorts. In Italy, the mean age of diagnosis is 23.9 [25], more than 10 years lower than the age of diagnosis in Israel, even though Israel has an overall younger population than Europe. These findings may indicate a low awareness of HS disease among patients and physicians leading to delayed diagnoses.

The co-morbidities of HS in our study correspond to previous reports [26].^.^ We found a high percentage of smokers, most of them males (66%), among patients. Additionally, we found a higher prevalence of obesity in HS patients compared to the general population.

Regarding family history, only 18% of patients had a positive family history of HS (20.4% among Jewish patients compared to 14% among Arab patients). This differs from the data found in the literature, suggesting that 30–40% of patients had a positive family history [27]. This finding could be unique to patients with more severe HS referred to our tertiary centre and/or genetic characteristics of Arab and Jewish patients in Israel. Genetic profiling of HS patients should be investigated in more depth.

According to our study, the body area that was the most involved was the groin, followed by the axilla and buttocks. This corresponds to the results of Revuz’s study showing that the groin, axilla, and perianal region are the most frequently affected areas [2]. We found that lesions in the axilla and buttocks are predisposing factors to severe HS. Schrader et al. [28] also demonstrated that the involvement of the axilla, buttocks, and breast regions increases the risk of severe disease, but statistical significance was shown only with univariate analysis (analysis of a single area in the body) and not with multivariate analysis of the different regions. In addition, we found that men who smoke and are overweight are predisposed to a more severe form of the disease. This corresponds to the results of Shalom et al. [22] as well as those of Canoui-Poinrine [24] and Schrader et al. [28]. On its own, ethnicity did not correlate with severe disease.

The treatment of HS disease is considered a great challenge for dermatologists. Topical and systemic antibiotics, oral retinoids, hormone therapy, corticosteroids, and biological treatments, mainly anti-TNF alpha and surgical or laser modalities, are the main treatment options and are used according to disease severity [29,30,31]. Adalimumab is the only treatment approved for the treatment of HS in moderate and severe cases and was found to be effective and well tolerated by patients with moderate to severe HS [32,33]. In our study, the response rates to adalimumab treatment were consistent with previous studies and showed good results: 41% of patients had marked improvement (8% of them had complete remission), 42% had partial improvement, and only 17% did not show any response. Both the latency of diagnosis and the location of the lesions affected the response to adalimumab: long latency and buttock lesions were the worst prognostic factors. However, it was found that ethnicity and sex did not affect the response. None of the adalimumab patients in this study had incidents of paradoxical psoriasiform, as described by Burzi, Lorenza et al. [34].

In summary, we found differences in the Arab and Jewish HS population, especially with respect to the high prevalence of the disease in the Arab population, mainly in Arab males. This could be attributed to their low socio-economic situation. On the other hand, this should be further explored using genetic studies to identify potential differences in predisposing mutations [35].

In addition, we show significant effectiveness of adalimumab treatment in most patients with moderate and severe HS in our cohort composed of Jews and Arabs. Response was better in patients with early diagnosis, emphasising its importance. This is especially critical for our patients, where we recognised the high mean age of diagnosis and the significant delays observed in diagnosis.

Increased awareness of HS among patients and physicians may result in earlier diagnoses, which, along with lifestyle adjustments, may improve the prognosis of HS patients. Future studies should address and answer the unmet need for better treatment options.

## 5. Limitations

The main limitation of this study is its retrospective nature. Statistical analysis of demographic and clinical influences on treatment response has some limitations: First, part of the collected data is based on personal statements from patients. Additionally, the combination of different treatments did not allow an accurate evaluation of each therapy as a single therapy. The clinical evaluation of the disease burden was not based on a recognised and accepted international score, such as ISH4 nor HiSCR; the only measure available was the Hurley scale. In addition, the differences in duration of treatment among patients may have affected treatment response. Another limitation of the study was that ultrasound examinations of HS lesions were not performed. This is a more sensitive method than clinical palpations and is currently the procedure of choice to evaluate improvement during anti-inflammatory treatments [36].

## Figures and Tables

**Table 1:** Demographic data and disease characteristics.

	N = 164 (%)
Gender	
Male	96 (58.5%)
Female	68 (41.5%)
Age at diagosis; range (years)	33.9 ± 13.8; 11–75
Ethnicity	
Jew	93 (57%, 44% adjusted)
Arab	71 (43%, 56% adjusted)
Hurley	
1	33 (20%)
2	83 (51%)
3	48 (29%)
Hypertension	5 (3%)
Overweight	46 (28%)
Hyperlipidaemia	34 (21%)
Diabetes mellitus	8 (5%)
Smoker	90 (55%)
Time until diagnosis (years)	4 [1–10]
Family history of HS	29 (18%)
Past medical history	57 (35%)
Affected body area	
Groin	122 (74%)
Axilla	103 (63%)
Buttocks	71 (43%)
Sub-mammary	44 (27%)
Associated condition	
Acne	18 (11%)
Eczema	20 (12%)
Psoriasis	11 (7%)
Others	10 (6%)

**Table 2:** Age of onset according to gender.

	N	Male	Female
No. of HS Patients	164	96 (58.5%)	68 (41.4%)
9–0 (year)	0	0	0
19–10 (year)	25 (15.2%)	10 (10.4%)	15 (22%)
29–20 (year)	50 (30.4%)	30 (31.25%)	20 (29.4%)
39–30 (year)	37 (22.5%)	16 (16.6%)	21 (30.8%)
49–40 (year)	26 (15.8%)	17 (17.7%)	9 (13.2%)
50+ (year)	26 (15.8%)	23 (23.9%)	3 (4.4%)

**Table 3:** Demographic data in relation to general population.

Gender			*p* < 0.0001
Male	96 (58.5%)	8270 (37.4%)	
Female	68 (41.5%)	13,815 (62.6%)	
Age; range	33.9 ± 13.8; 11–75	38.5 ±15.5	*p* < 0.0001
Ethnicity			*p* < 0.0001
Jew	93 (57%)	19,580 (88.7%)	
Other	71 (43%)	2505 (11.3%)	
Overweight	46 (28%)	1554 (7%)	*p* < 0.0001
Smoker	90 (55%)	2838 (12.9%)	*p* < 0.0001

Data of general population in Israel according to study conducted from patient data registered with Clalit Health Services [22].

**Table 4:** Treatment options and treatment response.

Treatment/Response	N = 164	Complete Remission	Significant Response	Mild Response	No Effect
Topical treatment	n = 156	-	-	15/156 (10%)	141/156 (90%)
		surgical treatment			
Laser deroofing	n = 7	7/7 (100%)	-	-	-
Wide excision	n = 24	-	2 (8%)	5 (21%)	17 (71%)
Surgical deroofing	n = 10	-	1 (10%)	4 (40%)	5 (50%)
Incision and drainage	n = 61	1 (2%)	7 (11%)	11 (18%)	42 (69%)
	systemic pharmaceutical treatment				
Dapsone	n = 5	-	2/5 (40%)	-	4/5 (60%)
Rifampin	n = 62	2 (3%)	8 (13%)	15 (24%)	37 (60%)
Clindamycin	n = 128	8 (6%)	27 (21%)	40 (31%)	53 (41%)
Augmentin/cephalosporin	n = 26	1 (4%)	1 (4%)	7 (27%)	17 (65%)
Minocycline	n = 29	1 (3%)	3 (10%)	5 (17%)	20 (69%)
Doxycycline	n = 29	-	2 (7%)	11 (38%)	16 (55%)
Systemic steroids	n = 24	-	1 (4%)	6 (25%)	17 (71%)
Acitretin	n = 27	1 (4%)	6 (22%)	2 (7%)	18 (67%)
Isotretinoin	n = 40	-	4 (10%)	10 (25%)	26 (65%)
Adalimumab	n = 36	3 (8%)	12 (33%)	15 (42%)	6 (17%)
Infliximab	n = 5	1 (20%)	1 (20%)	2 (40%)	1 (20%)

**Table 5:** Demographic data and disease characteristics with treatment response to Adalimumab.

N = 36	Complete Remission + Significant; n = 15 (1)	Mild; n = 15 (3)	No Effect; n = 6 (4)	*p*-Value
Ethnicity (Jews)	7 (47%)	10 (67%)	2 (33%)	*p* = 0.32
Arabs	8 (53%)	5 (33%)	4 (67%)	
Gender (male)	9 (60%)	12 (80%)	5 (83%)	*p* = 0.38
Female	6 (40%)	3 (20%)	1 (17%)	
Hurley				*p* = 0.78
1	0	1 (7%)	0	
2	4 (27%)	5 (33%)	2 (33%)	
3	11 (73%)	9 (60%)	4 (67%)	
Age (years)	38.7 ± 12.8	39.5 ± 14.6	39.8 ± 16.0	*p* = 0.98
Time until diagnosis (years)	2 [0.33–6.0]	6 [3–15]	5 [0.48–24.5]	1 vs. 3 *p* = 0.049
Hypertension	2 (13%)	1 (7%)	0	NA
Obesity	7 (47%)	5 (33%)	2 (33%)	*p* = 0.72
Hyperlipidaemia	3 (20%)	2 (13%)	1 (17%)	*p* = 0.88
Diabetes mellitus	2 (13%)	2 (13%)	0	*p* = 0.64
Smoker	7 (47%)	10 (67%)	3 (50%)	*p* = 0.52
Location (groin)	13 (87%)	11 (73%)	6 (100%)	*p* = 0.30
Axilla	12 (80%)	8 (53%0	5 (83%)	*p* = 0.21
Buttocks	7 (47%)	6 (40%)	6 (100%)	*p* = 0.037 1 vs. 4—0.045 3 vs. 4—0.02
Sub-mammary	9 (60%)	2 (13%)	2 (33%)	Small numbers
White blood cells	5 (33%)	3 (20%)	0	*p* = 0.24
Liver enzymes	2 (13%)	5 (33%0	0	*p* = 0.16

## Data Availability

All data generated or analysed during this study are included in this article. Further enquiries can be directed to the corresponding author.

## References

[1] Jemec G.B.E. (2012). Clinical practice. Hidradenitis suppurativa. N. Engl. J. Med..

[2] Revuz J. (2009). Hidradenitis suppurativa. J. Eur. Acad. Dermatol. Venereol..

[3] Gill L., Williams M., Hamzavi I. (2014). Update on hidradenitis suppurativa: Connecting the tracts. F1000Prime Rep..

[4] Fitzsimmons J.S., Guilbert P.R. (1985). A family study of hidradenitis suppurativa. J. Med. Genet..

[5] Lapins J., Jarstrand C., Emtestam L. (1999). Coagulase-negative staphylococci are the most common bacteria found in cultures from the deep portions of hidradenitis suppurativa lesions, as obtained by carbon dioxide laser surgery. Br. J. Dermatol..

[6] Roenigk R.K., Roenigk H.H. (1989). Axillary Hyperhidrosis, Apocrine Bromhidrosis, Hidradenitis Suppurativa, and Familial Benign Pemphigus: Surgical Approach.

[7] Rabasseda X. (2013). A report from the 22nd Congress of the European Academy of Dermatology and Venereology (October 2-6, 2013–Istanbul, Turkey). Drugs Today.

[8] Kraft J.N., Searles G.E. (2007). Hidradenitis Suppurativa in 64 Female Patients: Retrospective Study Comparing Oral Antibiotics and Antiandrogen Therapy. J. Cutan. Med. Surg..

[9] Lapins J., Ye W., Nyrén O., Emtestam L. (2001). Incidence of Cancer Among Patients With Hidradenitis Suppurativa. Arch. Dermatol..

[10] Maclean G.M., Coleman D.J. (2007). Three Fatal Cases of Squamous Cell Carcinoma Arising in Chronic Perineal Hidradenitis Suppurativa. Ann. R. Coll. Surg. Engl..

[11] Shavit E., Dreiher J., Freud T., Halevy S., Vinker S., Cohen A. (2014). Psychiatric comorbidities in 3207 patients with hidradenitis suppurativa. J. Eur. Acad. Dermatol. Venereol..

[12] Fimmel S., Zouboulis C.C. (2010). Comorbidities of hidradenitis suppurativa (acne inversa). Dermato-Endocrinology.

[13] Jemec GB E., Wendelboe P. (1998). Topical clindamycin versus systemic tetracycline in the treatment of hidradenitis suppurativa. J. Am. Acad. Dermatol..

[14] Gener G., Canoui-Poitrine F., Revuz J.E., Faye O., Poli F., Gabison G., Pouget F., Viallette C., Wolkenstein P., Bastuji-Garin S. (2009). Combination Therapy with Clindamycin and Rifampicin for Hidradenitis Suppurativa: A Series of 116 Consecutive Patients. Dermatology.

[15] Joseph M.A., Jayaseelan E., Ganapathi B., Stephen J. (2005). Hidradenitis suppurativa treated with finasteride. J. Dermatol. Treat..

[16] Boer J., Nazary M. (2011). Long-term results of acitretin therapy for hidradenitis suppurativa. Is acne inversa also a misnomer?. Br. J. Dermatol..

[17] Grant A., Gonzalez T., Montgomery M.O., Cardenas V., Kerdel F.A. (2010). Infliximab therapy for patients with moderate to severe hidradenitis suppurativa: A randomized, double-blind, placebo-controlled crossover trial. J. Am. Acad. Dermatol..

[18] Gupta A.K., Studholme C. (2016). Adalimumab (Humira) for the Treatment of Hidradenitis Suppurativa. Skin Ther. Lett..

[19] Mahmoud B.H., Tierney E., Hexsel C.L., Pui j Ozog D.M., Hamzavi I.H. (2010). Prospective controlled clinical and histopathologic study of hidradenitis suppurativa treated with the long-pulsed neodymium:yttrium-aluminium-garnet laser. J. Am. Acad. Dermatol..

[20] Mikkelsen P.R., Dufour D.N., Zarchi K., Jemec G.B. (2015). Recurrence rate and patient satisfaction of CO_2_ laser evaporation of lesions in patients with hidradenitis suppurativa: A retrospective study. Dermatol. Surg..

[21] Lapins J., Marcusson J.A., Emtestam L. (1994). Surgical treatment of chronic hidradenitis suppurativa: CO_2_ laser stripping-secondary intention technique. Br. J. Dermatol..

[22] Shalom G., Babaev M., Freud T., Tiosano S., Pam N., Horev A., Diriher J., Vardy D.A., Comaneshter D., Cohen A.D. (2017). Demographic and health care service utilization by 4417 patients with hidradenitis suppurativa. J. Am. Acad. Dermatol..

[23] Kurokawa I., Hayashi N. (2015). Japan Acne Research Society. Questionnaire surveillance of hidradenitis suppurativa in Japan. J. Dermatol..

[24] Canoui-Poitrine F., Revuz J.E., Wolkenstein P., Viallette C., Gabison G., Pouget F., Poli F., Faye O., Bastuji-Garin S. (2009). Clinical characteristics of a series of 302 French patients with hidradenitis suppurativa, with an analysis of factors associated with disease severity. J. Am. Acad. Dermatol..

[25] Bettoli V., Pasquinucci S., Caracciolo S., Piccolo D., Cazzaniga S., Fantini F., Binello L., Pintori G., Naldi L. (2016). The Hidradenitis supurativa patient journey in Italy: Current status, unmet needs and opportunities. J. Eur. Acad. Dermatol. Venereol..

[26] Kim W.B., Sibbald R.G., Hu H., Bashash M., Anooshirvani N., Coutts P., Alavi A. (2016). Clinical features and patient out-comes of hidradenitis suppurativa: A cross-sectional retrospective study. J. Cutan. Med. Surg..

[27] Von Der Werth J.M., Williams H.C., Raeburn J.A. (2000). The clinical genetics of hidradenitis suppurativa revisited. Br. J. Dermatol..

[28] Schrader A.M., Deckers I.E., Van Der Zee H.H., Boer J., Prens E.P. (2014). Hidradenitis suppurativa: A retrospective study of 846 Dutch patients to identify factors associated with disease severity. J. Am. Acad. Dermatol..

[29] Gulliver W., Zouboulis C.C., Prens E., Jemec G.B., Tzellos T. (2016). Evidence-based approach to the treatment of hidradenitis suppurativa/acne inversa, based on the European guidelines for hidradenitis suppurativa. Rev. Endocr. Metab. Disord..

[30] Sbidian E., Hotz C., Seneschal J., Maruani A., Amelot F., Aubin F., Paul C., Barry M.B., Humbert P., Dupuy A. (2016). Antitumour necrosis factor-alpha therapy for hidradenitis suppurativa: Results from a national cohort study between 2000 and 2013. Br. J. Dermatol..

[31] Martin-Ezquerra G., Masferrer E., Masferrer-Niubo M., Ferran M., Sánchez-Regaña M., Collgros H., Bordas X., Notario J., Alsina M., Gil I. (2015). Use of biological treatments in patients with hidradenitis suppurativa. J. Eur. Acad. Dermatol. Venereol..

[32] Kimball A.B., Okun M.M., Williams D.A., Gottlieb A.B., Papp K.A., Zouboulis C.C., Armstrong A.W., Kerdel F., Gold M.H., Forman S.B. (2016). Two Phase 3 Trials of Adalimumab for Hidradenitis Suppurativa. N. Engl. J. Med..

[33] Kim E.S., Garnock-Jones K.P., Keam S.J. (2016). Adalimumab: A Review in Hidradenitis Suppurativa. Am. J. Clin. Dermatol..

[34] Burzi L., Repetto F., Ribero S., Mastorino L., Quaglino P., Dapavo P. (2022). Paradoxical psoriasiform reactions during treatment with adalimumab for hidradenitis suppurativa: Real-life experience and therapeutic response to other biological drugs. Dermatol. Ther..

[35] Pink A.E., Simpson M.A., Brice G.W., Smith C.H., Desai N., Mortimer P.S., Barker J.N., Trembath R.C. (2011). PSENEN and NCSTN mutations in familial hidradenitis suppurativa (Acne Inversa). J. Investig. Dermatol..

[36] Nazzaro G., Passoni E., Guanziroli E., Casazza G., Muratori S., Barbareschi M., Veraldi S., Marzano A.V. (2018). Comparison of clinical and sonographic scores in a cohort of 140 patients with hidradenitis suppurativa from an Italian referral centre: A retrospective observational study. Eur. J. Dermatol..

